# Supplementary material for: Splice-Junction-Based Mapping of Alternative Isoforms in the Human Proteome
Source: Cell Rep. Author manuscript; Available in PMC 2020 Jan 15. (PMC6961840; doi:10.1016/j.celrep.2019.11.026)
Supplement: 2 [file NIHMS1546469-supplement-2.zip › DF1/PXD006675/LeftCoronaryArtery/LeftCoronaryArtery_10_FLNA_FGGEHVPNSPFQVTAPER.pdf]

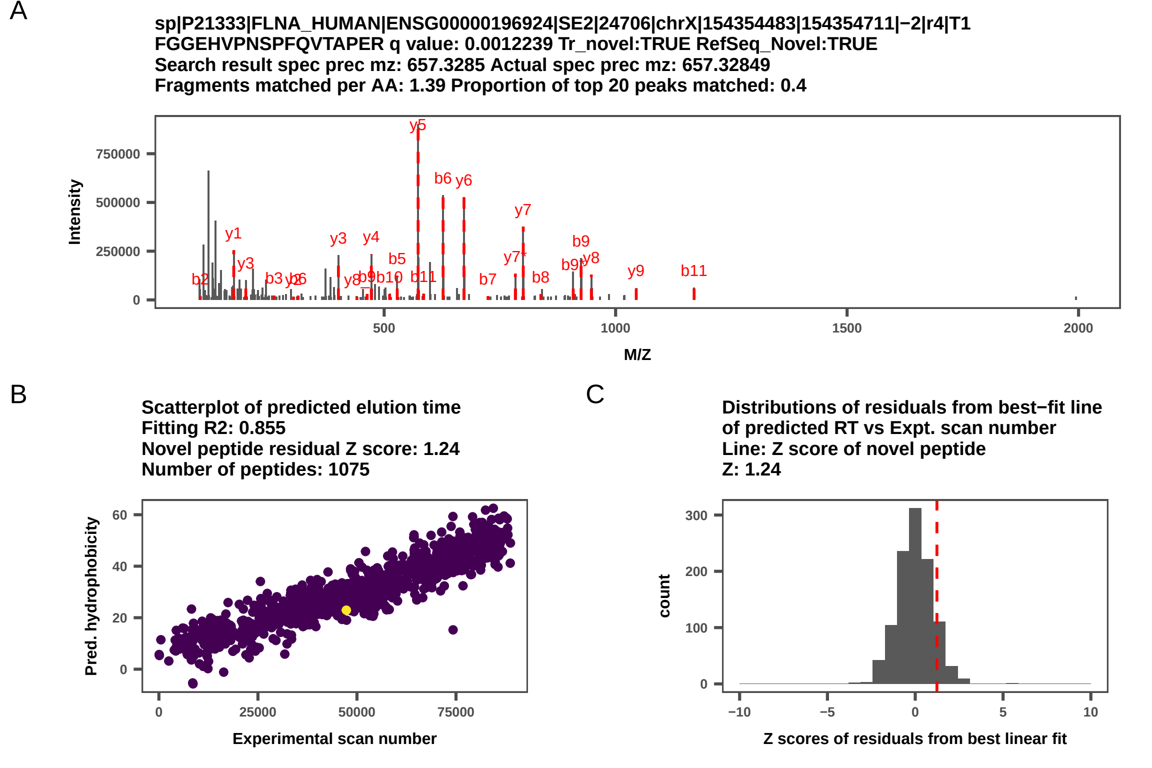
A
sp|P21333|FLNA_HUMAN|ENSG00000196924|SE2|24706|chrX|154354483|154354711|−2|r4|T1
FGGEHVPNSPFQVTAPER q value: 0.0012239 Tr_novel:TRUE RefSeq_Novel:TRUE
Search result spec prec mz: 657.3285 Actual spec prec mz: 657.32849
Fragments matched per AA: 1.39 Proportion of top 20 peaks matched: 0.4
Intensity
750000
500000
250000
0
500
1000
1500
2000
M/Z
y5
b6
y6
y7
y1
y3
y4
b9
y3
b5
y7*
b9*
y8
y9
b11
b2
b3
y2
b6
y8
b9
b10
b11
b7
b8
B
Scatterplot of predicted elution time
Fitting R2: 0.855
Novel peptide residual Z score: 1.24
Number of peptides: 1075
Pred. hydrophobicity
60
40
20
0
0
25000
50000
75000
Experimental scan number
C
Distributions of residuals from best−fit line
of predicted RT vs Expt. scan number
Line: Z score of novel peptide
Z: 1.24
count
300
200
100
0
−10
−5
0
5
10
Z scores of residuals from best linear fit
